# A Case of Successful Employment of the Rhus Toxicodendron in Paralysis

**Published:** 1830

**Authors:** John Eberle


Art. V.—*A case of the successful employment of the Rhus Toxicodendron in paralysis.*—By John Eberle, M. D., &c. &c.

Miss O. aged about 47, of a spare habit of body, and nervous temperament, was suddenly seized on the 17th of June, 1827, with vertigo, nausea, great debility and total loss of voluntary motion in the whole left side of her body. I saw her an hour after the commencement of the attack, and found her with a pale and anxious countenance; a small, rather tense, and quick pulse; pupils contracted; skin of the natural temperature on both sides; and remarkably loquacious, though incapable of distinct articulation. The sensibility of the affected side was not in the least degree impaired. There was not the slightest somnolency—on the contrary an almost constant wakefulness tormented the patient for many days. I prescribed, at first, an active mercurial purgative, which operated very freely; frictions with strong tincture of capsicum and cupping along the spine and back of the neck. Blisters were afterwards applied to the wrist and ankle of the affected extremities. I continued this treatment with an occasional laxative about ten days, without deriving the least advantage from it. I then prescribed the saturated Tincture of the *Rhus Toxicodendron* in doses of 40 drops three times daily. On the third day after commencing with this remedy, stiong involuntary starts of the muscles of the affected side occurred, and on the following day the muscles of the fingers were under the command of the will. Under the employment of this medicine, without any other remediate measures, the empire of volition gradually extended itself, until in the course of about ten days the muscles of the affected arm were fully under its command.

The muscles of the leg however, still remained inobedient to the calls of the will. The use of the tincture of *rhus* was now discontinued and frictions, mustard seed internally, and blistering along the lower part of the spine, again resorted to. At the end of eight days, the patient's condition was not in the least improved. The tincture of Rhus was therefore resumed, in doses of 45 drops thrice daily. In the course of four days from the resumption of the medicine, strong involuntary contractions of the affected limb again ensued; and in a few days more, the patient could slightly move the thigh on the hip; but over the motions of the leg, ankle and toes she had not the least command. In a very short time however, she found herself able to move the whole limb; and she is now (August 7,) capable, with a little aid, to walk in her chamber. She experiences daily a manifest increase of muscular power in the leg. The powers of the arm are completely restored.

It is somewhat singular that the return of voluntary motion in the *upper* extremity commenced in the first joints of the fingers, and was gradually extended upwards to the shoulder; whereas in the lower extremity, voluntary motion was first manifest in the power of moving the thigh on the hip, then successively passing downwards,—the ankle and the toes remaining longest in a paralytic condition.

The Rhus Toxicodendron is by no means a novel, although at present an almost wholly neglected remedy. Dufresney, Kruger, Elz, Alderson, Horsefield, and others have adduced much evidence of its remediate powers in paralysis. Besides the case related above, I have seen another instance of its usefulness in this affection. From a letter which I received from Professor Osann of Berlin, I learn that in Germany this medicine has within the last three or four years been employed with marked success in paralytic affections.

